# The study of nurses’ knowledge and attitudes regarding pain management and control in emergency departments

**DOI:** 10.1186/s12873-023-00793-y

**Published:** 2023-03-13

**Authors:** Sajjad Ahmadi, Parya Vojdani, Hamid Reza MortezaBagi

**Affiliations:** Emergency Medicine Research Team, Tabriz University of Medical Sciences, Tabriz, Iran

**Keywords:** Knowledge, Attitude, Nurse, Emergency, Pain management

## Abstract

**Background:**

Given the importance and pivotal role of nurses in pain management and control, this study was conducted to determine the nurses’ knowledge and attitudes toward in emergency departments.

**Methods:**

This study was designed and conducted as a descriptive-analytical cross-sectional study. Nurses’ attitude and knowledge towards pain management and control and relationship between their demographic characteristics have been assessed. Nurse Attitude Survey (NAS) and Pain management and control principles assessment Test (PMPAT) questionnaires were used.

**Results:**

Totally 400 volunteers, including 148 (37.2%) male and 250 (62.8%) female nurses recruited from 23 hospitals in East Azerbaijan, Iran, with a mean age of 30.88 years (± 6.04 SD) and age range between 22 and 53 years old. The crude mean score of participants’ knowledge of pain management and control was 12.51 (± 2.77 SD), and standardized mean score was 40.34 (± 8.92 SD), which was low at 84.8% and moderate in 15.3% of the participants. Older nurses and nurses who previously participated in pain retraining courses had significantly less knowledge about pain management and control (r= -0.104, P = 0.038), and (r= -0.148, P = 0.003) respectively. The crude mean score of participants’ attitudes toward pain control and management was 15.22 (± 2.56 SD), and standardized mean score was 60.87 (± 10.26 SD). Nurses’ attitudes have become more negative with the increase of their work experience (r = -0.168, P = 0.001), and previously participation in pain retraining courses (r =-0.207, P < 0.001). Older nurses and highly educated nurses had significantly more negative attitudes towards pain control and management (r = -0.153, P = 0.002), and (r= -0.126, P = 0.005), respectively.

**Conclusions:**

The current study revealed that pain management and control knowledge in most emergency nurses was low, and most of them had a moderate attitude. We need more scientific and comprehensive pain management and control training courses to improve knowledge and attitude among health workers and nurses.

## Introduction

Caused by actual or potential tissue damage; pain is an unpleasant sensory-emotional [1, 2], and is among one of the most common reasons for referring patients to medical centers [3]. Recognizing and treating pain is one of the oldest sciences that human kind has been trying to study and perfect since the beginning of creation with a continuous and tireless effort, and of course, it has led to exciting and impressive achievements. [1, 2].Approximately, 76 million adults in the United States suffer from pain, and a lack of properly control and manage pain can be costly for the family and society. In the United States, the annual cost of pain is estimated at $ 635 billion [4]. According to estimates made by the Iranian Rehabilitation and Electro diagnosis Association, 10–20% of Iranians(about 1 out of every 6 people) suffer from some types of chronic pain, and as the elderly population increases, this figure may increase significantly to 30.% [5].

A survey in Tehran showed that 25.5% of people suffer from chronic pain, which the prevalence rate of chronic pain in married, housewives, retired, and pensioners was much more than other social groups and showed the role of patterns such as age, educational status, depression and anxiety in suffering from chronic pain [6]. However another study that was conducted among the main working groups including tools and equipment, organizational aspects, environment, time aspects of the job, biomechanics, individual characteristics and education, showed that the prevalence of pain during life in Iran was about 70% [5, 7, 8]. The results showed that 41% of all participants were workers in industrial occupational group followed by workers in health sector (28%) the highest prevalence of LBP is pertained to agriculture (58.22%) and handicrafts (58.88%), respectively, and the lowest is related to music (18.50%) [6, 7].

In addition to the financial aspect, unrelieved and long-term pain leads to physiological and psychological complications [9]. Patients with chronic pain should always evaluated for physically and psychologically aspects of their pain, because pain affect their quality of life. Chronic pain interferes with patients’ daily activities, leading to isolation and withdrawal from family and friends, reduced working hours, lost workdays and so has a detrimental economic impact [10]. The psychological effects of pain can cause depression, anxiety, aggression, reduced independence, and disruption in interpersonal relationships [11, 12]. It can also cause unnatural fears, worries about the future, and adverse effects on family dynamism [13, 14]. The physiological effects of unrelieved pain can manifest in various organs, including cardiovascular, gastrointestinal, genitourinary system, musculoskeletal, and immune systems [15].

Since nurses spend more time with the patient than other treatment staff, they should be well trained and familial with pain management and control [16]. Nurses possess a unique position that directly impacts the patient’s pain management and control process, from evaluation, planning, to intervention and reassessment [9]. Nurses constantly need to make decisions concerning controlling patients’ pain; however effective control requires the proper decision-making process that is possible through awareness of pain and its treatment [17]. The usefulness of pain assessment includes obtaining basic information for subsequent assessments, diagnosing the degree of disability with defective treatment outcomes, assisting the physician in diagnosing the special condition, discerning between the actual pains and malingering as well as improving the patient-physician relationship [18, 19].

However, few studies in our country show that nurses do not have enough information in this regard which indicated that the majority of nurses have not received any pain training assessment during their academic courses [18, 20–23]. Considering the importance and prevalence of pain and the pivotal role of nurses in managing and controlling pain, providing patient comfort and lack of sufficient related studies in Iran, present study was conducted with the aim of determining the knowledge and attitude of nurses in the emergency department.

According to “The International Association for the Study of Pain” definition of “Pain” is an unpleasant sensory and emotional experience, associated with or expressed in terms of actual or potential tissue damage or damage to another tissue type [24]. “Knowledge” can be defined as facts, information, and skills acquired through experience or education; the theoretical or practical understanding of a subject; ability to know and understand or have knowledge about events [25] However “Attitude” is a combination of beliefs and emotions that prepare a person in advance to look at others, objects, and groups positively or negatively [26].

## Methods

### Research design

This study was designed and conducted as a descriptive-analytical cross-sectional study. The target research population was nurses working in emergency departments of hospitals affiliated with Tabriz University of Medical Sciences to study their knowledge and attitudes towards pain management and control. Therefore, the following aims were considered:

1. Determining the score of nurses ‘knowledge and attitudes toward pain management and control;
2. Investigating the relationship between nurses’ attitude scores regarding pain management and control and their demographic characteristics;
3. Investigating the relationship between nurses’ knowledge scores regarding pain management and control and their demographic characteristics.

#### Study subjects and population (setting and sampling)

The sample size for the current study was calculated using the information reported for the knowledge and attitude score in the study of Aflatoonian et al. [27], and the maximum sample size was considered.

In the sample size calculation formula used, the alpha error was considered 5%, the mean of the sample was equal to 64.14, and the standard deviation was 32.7. Finally, with an accuracy of 36.1, the total sample size was measured as 230 subjects.

We conducted probability sampling, which means every member of the population has a chance to be selected. Based on the gathered information, approximately 1200 nurses were working in the emergency departments of the province’s hospitals and the number of nurses varied among hospitals. All these ratios have been obtained for different hospitals and have been sampled accordingly. Every study subject was listed with a number using systematic sampling, but instead of randomly generating numbers, individuals were selected at regular intervals. After explaining the objectives of the research and its voluntary nature, all participants completed the pertinent questionnaires and they were collected.

### Inclusion and exclusion criteria

All nurses working morning, evening, and night shifts who agreed to voluntarily and actively participate in the study were included in the study. However, nurses who did not agree to participate, or were not present during the data collection period, due to any reason were excluded from the study.

### Data collection and instruments

The research questionnaire tool consisted of three parts:

- Nurses’ demographic information checklist included age, gender, marital status, education, employment status, work experience, place of work, passing a training course related to pain management and control;
- Nurses’ attitudes toward pain management and control as Nurse Attitude Survey (NAS) questionnaire; and.
- Nurses’ Knowledge Assessment Questionnaire as Pain management and control principles assessment Test (PMPAT).

McMillan designed both questionnaires to assess nurses’ attitudes and knowledge toward pain management and [28].The Attitude Questionnaire consists of 25 questions in which the respondent selects an option based on their opinion. Likert scoring system (strongly disagree, disagree, agree, and strongly agree) was used, with a score of 1,2,3,4, respectively. Higher scores indicate a positive attitude. If the respondent receives 70% or higher, s/he has the highest and most positive attitude score; receiving 50–70% of the score indicates an average attitude level, and less than 50% shows a negative attitude.

The knowledge assessment questionnaire (PMPT) consists of 31 four- and five- multiple-choice questions, which assess the individual’s level of knowledge concerning the concept of pain, pain assessment, pain relief methods, and analgesics. The correct option according to the individual’s opinion was marked. Each correct answer was given a score. Scores ranged from 0 to 31, or 0 to 100% [29]. Both of questionnaires have been translated into Persian by the research team at Jiroft Nursing School. Also, its validity and reliability was performed on the basis of internal correlation coefficient using Cronbach’s alpha (86%) [27].

### Statistical analysis

Descriptive statistics were used as descriptive tables and indicators, including mean and standard deviation to describe demographic characteristics, as well as nurses’ knowledge and attitudes scores toward pain management and control.

Inferential statistics in the form of Spearman’s correlation coefficient test were used to show the relationship between knowledge and attitude, and also to determine the relationship and association between of nurses’ knowledge and attitudes with their demographic characteristics and their concerning scores, using student T-test and analysis of variance (ANOVA). The significance level for all tests was less than 0.05, and with 95% confidence intervals (CIs).

Correlation coefficient was used as indicators of the strength of the assessment of linear relationship between different variables and Knowledge and Attitude scores. A linear correlation coefficient that is greater than zero indicates a positive relationship. A value that is less than zero signifies a negative relationship. Correlation coefficient r < 0.2 is a very weak correlation; r = 0.2 − 0.39 a weak correlation; r = 0.4 − 0.59 is a moderate correlation; r = 0.6 − 0.79 is a strong correlation and r = 0.8 − 1 is a very strong correlation. All data were calculated using STATA MP 14.2 (Stata Corp LP, College Station, Texas 77,845 USA).

## Results

Socio-demographic Characteristics of the Study Participants.

Totally 400 volunteers, including 148 (37.2%) male and 250 (62.8%) female nurses recruited for this study from emergency departments of 23 hospitals in East Azerbaijan, Iran. They were between 22 and 53 years old with a mean age of 30.88 years (± 6.04 SD). Of these 265 (66.3%) nurses were married, 371 nurses (92.8%) were undergraduate, and 26 nurses (6.5%) were postgraduate.

Most the participant nurses (80.4%) had less than ten years of work experience, and just13 nurses (3.3%) had over twenty years of work experience. 316 nurses (79%) worked under contracted and just 155 nurses (38.8%) had previously participated in pain management and control continuous education training courses. Descriptive summary results of participating nurses were presented in Table 1.

**Table 1:** Summary of demographic results of 400 participants, and Knowledge and Attitude of Emergency ward Nurses In affiliated Hospitals of Tabriz University of Medical sciences about pain management

Variable		Frequency	Percentage	Knowledge Scores		Attitude Scores	
				Mean (± SD)	P	Mean (± SD)	P
Sex	Male	148	37.2	39.49(9.34)	0.199	63.62(4.17)	0.160*
	Female	250	62.8	40.67(8.49)		63.02(4.05)	
Marriage Status	Single	114	28.9	40.80(8.20)	0.079	63.81(3.99)	0.035**
	Married	265	67.1	40.23(9.12)		62.97(4.14)	
	Widow	1	0.3	35.48		54	
	Divorced	15	3.8	34.62(7.46)		63.73(3.88)	
Education	Bachelor	371	93.5	40.44(8.93)	0.305	63.39(3.99)	0.009*
	MA	26	6.5	38.59(8.58)		61.23(5.36)	
Work Experience(Year)	< 3	95	23.9	42.04(7,48)	0.244	64.33(4.08)	0.001**
	4–7	160	40.2	39.62(9.04)		63.31(3.72)	
	8–11	65	16.3	40.40(9.25)		62.03(4.00)	
	12 − 5	40	10.1	40.65(11.52)		63.15(4.66)	
	16–19	25	6.3	38.71(8.59)		63.44(4.52)	
	> 20	13	3.3	37.47(5.97)		60.08(4.29)	
Employment	Un-Formal	84	21.4	41.55(7.68)	0.386	63.68(4.01)	0.543**
	Contractual	157	40.1	39.94(8.69)		63.16(3.82)	
	Formal	151	38.5	40.21(9.63)		63.09(4.47)	
Participate in pain management courses	Yes	155	39.1	38.86(9.29)	0.004	62.2(4.19)	< 0.001*
	No	241	60.3	41.49(8.45)		63.9(3.89)	

* Results were reported using the T Independent test

** Results were reported using ANOVA test

### Knowledge scores about pain control and management

The crude mean score of participants’ knowledge of pain control and management was 12.51 (± 2.77 SD) with a range of 5 to 20 crude scores. The standardized mean score was 40.34 (± 8.92 SD) with a minimum of 16.13 and a maximum of 64.52 scores.

In total, the knowledge score was low at 84.8% and moderate in 15.3%of the participants. None of the participants had a high level of knowledge (more than 70 scores).Table 2 shows the whole questions and mean scores of the Pain management and control principles assessment Test (PMPAT) questionnaire in detail.

**Table 2:** Pain Management Principles Assessment Test (PMPAT) Questionnaire Results in 400 Nurses

No	Question	Response	Correct Answers Number (%)	Mean Score (± SD)
1	Percentage of cancer patients who experience pain at some point during their illness	90%	11(27.8)	0.28(0.45)
2	Percentage of cancer patients who suffer pain for longer than a month	80 − 70%	117(29.3)	0.29(0.46)
3	If the patient still complains of pain despite receiving the maximum dose of medication to relieve the pain, what should the nurse always do?	Contact with Physician	239(59.8)	0.60(0.49)
4	What is the best way to prescribe narcotic analgesics in cancer patients?	Oral	348(87.0)	0.87(0.34)
5	What is the best time to seek medication in a patient on prescription analgesia to relieve cancer pain?	Before the pain recurred	50(12.5)	0.13(0.33)
6	Who can make the most accurate and reliable judgment about the pain intensity of cancer patients?	patients	265(66.3)	0.66(0.47)
7	What percentage of patients who take narcotic analgesics on a scheduled basis become addicted to these drugs?	Less than 1%	-	-
8	Which of the following statements correctly describes the mechanism of action of analgesics?	Narcotic analgesics in the brain reduce pain transmission and perception	221(55.3)	0.55(0.50)
9	What type of pain can be treated with skin irritations?	Any severity of pain	269(67.3)	0.67(0.47)
10	Which of the following statements correctly describes the philosophy of using analgesics in patients with advanced cancer?	Long-term use leads to tolerance and may require higher doses to control pain.	161(40.3)	0.40(0.49)
11	Which group of symptoms is most associated with chronic pain?	Decreased appetite, decreased energy, sleep disorders, apathy, hypotension	135(33.8)	0.34(0.47)
12	Which of the following drugs has the longest duration of action?	Methadone	220(55.0)	0.55(0.50)
13	Which cases are usually associated with acute pain?	Increased caloric requirement and increased temperature	148(37.0)	0.37(0.48)
14	Which of the following is perceived as itching and throbbing pain?	C Fibers	84(21.0)	0.21(0.41)
15	According to pain control gate theory, what is the area responsible for this function in the nervous system?	Gelatinous fluid of the spinal cord	209(52.3)	0.52(0.50)
16	By what is pain regulated?	µ, Gamma and kappa opioid receptors	327(81.8)	0.82(0.39)
17	Ms. Colton is a 72-kilogram, 24-year-old woman. She underwent hysterectomy and dropped eight milligrams of morphine at 4 p.m. It is now 32:16 and he has complained of pain and is asking for more drugs. What is his pain most related to?	Reduce analgesic blood levels	110(27.5)	0.28(0.45)
18	What should be your goal in managing pain in Ms. Calton after an abdominal hysterectomy?	Complete painlessness	126(31.5)	0.32(0.47)
19	Mr. West has prostate cancer that has metastasized to the bone. What is the first factor we need to consider when caring for him?	Overall quality of life of Patient	197(49.3)	0.49(0.50)
20	In assessing the patient’s pain, what variables should the nurse consider that are effective in expressing pain?	A, B, C	236(59.0)	0.59(0.49)
21	How does naloxone work?	Opioid Antagonist	111(27.8)	0.28(0.45)
22	The researchers showed that:	Doctors prescribe less and nurses give less pain medication	70(17.5)	0.18(0.38)
23	What is one of the main disadvantages of meperidine?	Central neurological complication more than morphine	226(56.5)	0.57(0.50)
24	Which of the following methods of prescribing narcotic analgesics produces a fixed level of analgesia in the patient?	Intravenous opioids	55(13.8)	0.14(0.34)
25	What are the primary benefits of having a consistent level of pain control?	More patient comfort	141(35.3)	0.35(0.48)
26	The nurse’s decision to give analgesics should be based on all of the following, other than:	Objective nurse assessment of pain intensity	22(5.5)	0.06(0.48)
27	Who has the most control over the patient’s pain management program?	Patient	118(0.46)	0.30(0.46)
28	What is the meaning of this definition? After repeated doses of analgesic drugs, the effect of a drug decreases and the patient needs more and more doses of the drug. This decline begins with a reduction in the pain relief period first and then a reduction in the analgesic effect.	Tolerance	164(41.0)	0.41(0.49)
29	Ms. Stone has metastatic breast cancer and painful lesions in the spinal cord. Because of her fear of drugs, she prefers to use painkillers when needed for PRN. You massage her back and use a hot pack. This is an example of:	Cutaneous Irritation	68(17.0)	0.17(0.38)
30	Another approach you might consider about Ms. Stone. Focus on techniques such as working with tables, reading books, or knitting. What is this method called?	Intention Distract	198(49.5)	0.50(0.50)
31	Ms. Strick is a 72-year-old woman with cancer that has metastasized to the pelvis. In addition, she has severe arthritis. Which of the following is sufficient to manage her pain?	The use of morphine and a non-steroidal anti-inflammatory drug gives the best results with the least side effects.	252(63.0)	0.63(0.48)

The assessment of nurses’ knowledge scores and demographic characteristics using the Spearman correlation coefficient test revealed that pain control and management knowledge of participant nurses had a significant relationship with age and previous participation experience in pain control and management training courses. While older nurses had significantly less knowledge about pain control and management (r= -0.104, P = 0.038), and nurses who previously participated in pain retraining courses had significantly less knowledge of pain control and management (r= -0.148, P = 0.003) (Table 4).

### Attitude scores about pain control and management

The crude mean score of participants’ attitudes toward pain control and management was 15.22 (± 2.56 SD) with a range of 5 to 20 crude scores. The standardized mean score was 60.87 (± 10.26 SD), with a minimum of 36 and a maximum of 82 scores. Table 3 shows the whole questions and mean scores of the Nurses’ attitudes toward pain management and control as Nurse Attitude Survey (NAS) questionnaire results (Table 3).

**Table 3:** Nurses’ attitudes toward pain management and control as Nurse Attitude Survey (NAS) questionnaire Results in 400 Nurses

No	Question	Response, Numbers (%)				Scores Mean(± SD)
		Completely Disagree	Disagree	Agree	Completely Agree	
1	Giving opioids on a regular schedule is preferred over prn	39(9.8)	38(9.5)	219(54.8)	104(26)	0.74(0.22)
2	A patient should experience discomfort prior to receiving the next dose of pain medication	68(17.0)	84(0.21)	195(48.8)	52(13.0)	0.61(0.23)
3	Continuous assessment of pain and medication effectiveness is necessary for good pain management	39(9.8)	74(18.5)	166(41.5)	121(30.3)	0.73(0.23)
4	Patients (or their family members) have the right to request pain medication before the pain returns	34(9.3)	112(28.0)	125(31.3)	126(31.5)	0.71(0.24)
5	Patients (or family members) may be hesitant to ask for pain medication due to fear of the use of opioids	89(22.3)	161(40.3)	91(22.8)	58(14.5)	0.57(0.24)
6	Patients receiving opioids on a prn basis are likely to develop clock watching behaviors	80(20.0)	111(27.8)	170(42.5)	39(9.8)	0.61(0.23)
7	Estimation of pain by a physician or nurse is a more valid measure of pain than by patient report	125(31.3)	195(48.8)	58(14.5)	22(5.5)	0.76(0.21)
8	Patients in pain can tolerate high doses of opioids without sedation or respiratory depression	83(20.8)	257(64.3)	18(4.5)	42(10.5)	0.51(0.20)
9	Patients can be maintained in a pain free state	69(17.3)	277(69.3)	36(9.0)	18(4.5)	0.50(0.17)
10	If a patient reports pain relief and euphoria a lower dose of pain medication should be given the next time	69(17.3)	277(69.3)	36(9.0)	18(4.5)	0.72(0.23)
11	Patients with chronic pain should receive pain medication at regular intervals with or without the presence of pain	111(27.8)	139(34.8)	131(32.8)	18(4.5)	0.53(0.22)
12	Patients receiving around the clock opioids are at risk for sedation and respiratory depression	82(20.5)	163(40.8)	125(31.3)	28(7.0)	0.68(0.22)
13	Patients with severe chronic pain need higher doses of pain medication compared to acute pain	73(18.3)	194(48.5)	98(24.5)	33(8.3)	0.53(0.22)
14	Patients should be maintained in a pain free state	50(12.5)	91(22.8)	218(54.5)	39(9.8)	0.65(0.21)
15	Lack of pain expression does not necessarily mean lack of pain	14(3.5)	102(25.5)	208(52.0)	70(17.5)	0.70(0.20)
16	Cancer pain can be relieved with anti -cancer drugs, radiation therapy, and/or pain medications	87(21.8)	172(43.0)	125(31.3)	16(4.0)	0.54(0.20)
17	If the patient continues to have pain after receiving pain medication the nurse should contact the physician	35(8.8)	51(12.8)	225(56.3)	89(22.3)	0.73(0.21)
18	Patients receiving pain medications around the clock for cancer are likely to become addicted	41(10.3)	73(18.3)	130(32.5)	155(38.8)	0.50(0.25)
19	Distraction and diversion can decrease the perception of pain	50(12.5)	92(23.0)	180(45.0)	75(18.8)	0.67(0.24)
20	A constant level of analgesic should be maintained in the blood to control pain effectively	120(30.0)	108(27.0)	135(33.8)	36(9.0)	0.55(0.25)
21	Increasing analgesic requirements and physical symptoms are signs of addiction	55(13.8)	132(33.0)	181(45.3)	31(7.8)	0.63(0.21)
22	The cancer patient and family should have more control over the schedule for analgesics than the health professional	77(19.3)	131(32.8)	138(34.5)	54(13.5)	0.61(0.24)
23	The nurse can make a more accurate assessment of the patients pain than the patient	101(25.3)	146(36.5)	124(31.0)	29(7.2)	0.70(0.23)
24	Cutaneous stimulation (heat, massage, ice) are only effective for mild pain	54(13.5)	87(21.8)	165(41.3)	84(21.0)	0.56(0.25)
25	When is the best time to seek medication for a patient on cancer who is on prescription pain medication?	138(34.5)	171(42.8)	16(4.0)	68(17.0)	0.73(0.28)

The assessment of nurses’ attitude scores and their demographic characteristics using the Spearman correlation coefficient test revealed a statistically significant association between attitude scores and nurses ‘work experience. Simultaneously, it was found that nurses’ attitudes have become more negative with the increase of their work experience (r = -0.168, P = 0.001). Nurses who participated in pain retraining courses had a more negative attitude toward pain control and management (r =-0.207, P < 0.001). Older nurses and highly educated nurses had significantly more negative attitudes towards pain control and management (r = -0.153, P = 0.002), and (r= -0.126, P = 0.005), respectively. Furthermore, the study of the relationship between nurses’ knowledge and attitudes did not indicate a significant association (r = -0 / 039, P = 0/432) (Table 4).

**Table 4:** Summary of the results of association between Knowledge and Attitude of 400 participated nurses with sociodemographic variables

Variable	Attitude		Knowledge	
	P	r	P	r
Sex	0.161	-0.070	0.247	0.058
Age	0.002	-0.153	0.038	-0.104
Marital Status	0.107	-0.081	0.079	-0.089
Education	0.005	-0.126	0.178	-0.068
Work Experience	0.001	-0.168	0.052	-0.098
Work Status	0.372	-0.045	0.395	-0.043
Participation in Training Course	< 0.001	-0.207	0.003	-0.148
Hospitals of Tabriz	0.347	0,062	0,012	-0.166
City	0.072	-0.090	0.022	-0.115

## Discussion

This study aimed to evaluate the knowledge and attitudes of emergency nurses toward pain control and management. The results indicated that the knowledge of nurses concerning pain management and control seemed inadequate40.34 (± 8.92 SD) in the emergency departments in East Azerbaijan province hospitals, and 84.8% of them had insufficient knowledge. Pain control and management knowledge in participant nurses had a significant relationship with age and previous participation experience in pain control and management training courses. The attitude of nurses in pain management and control was 60.87(± 10.26 SD), and there was a statistically significant association between attitude score and nurses ‘work experience, while it was found that nurses’ attitudes have become more negative with the increasing of their work experience ( r = -0.168, P = 0.001).

The American Pain Association identified pain as the fifth vital sign needed for evaluation [30]. Although pain management and control is a priority in the care program, it is still a complex multi-dimensional problem involving medical, legal, socio-economic, and psychological aspects. However the effect of awareness and attitude factors is continuous in terms of concerning pain management and control. The first step in pain management and control is to evaluate patients’ pain [31], which can be measured using tools such as self-reporting systems, behavioral observation, and physiological measurements [32, 33].In the later stages, depending on the patient’s condition, various pharmacological and non-pharmacological methods are used to relieve the patient’s pain [34–36].

Three primary barriers hamper proper pain management and control that include: the perception of pain by medical staff, patient’s perception of pain, health care system management [13, 14]. Among the various factors, the medical staff’s knowledge and negative attitudes are crucial barriers to proper pain management and control [37].Among studies of physicians, nurses, and pharmacists, different results were obtained regarding pain perception and barriers to proper pain management and control [37–39].

In the case of physicians, time constraints, patient and companion attitudes, and fear of incorrect treatment were considered the main barriers to pain management and control [38]. Regarding nurses, patient and companion attitudes, physician’s performance and attitudes, and patients’ cultural differences were considered obstacles. Moreover, in the case of pharmacists, prescribing attitudes, time constraints, and lack of awareness were significant barriers [39].

Patients’ misperception of pain can occur in the form of non-reporting of pain due to misconceptions about treatment and side effects, fear of disease progression, and fear of painful treatment [40]. The treatment system itself is also an influential factor in managing patients’ pain, such as lack of staff awareness, unavailability of instructions, and lack of facilities. Insufficient budget and funding in this area and the existence of some restrictive rules can be an obstacle in the effective control and management of patients’ pain [37].

The type of nurses’ attitudes toward pain is another factor influencing the assessment and management of pain, that reflects their feelings, beliefs, and moods caused by excitement and express their ideas and beliefs. Simply put, they may easily be affected by challenging conditions and become apathetic or attentive in pain management and control based on their ideas and beliefs [41, 42].

The actions and behavior of nurses in care, including attention to the category of patients’ pain, may be influenced by various factors such as beliefs, values, customs, and economic status and, in general, can be influenced by the culture of the society, which leads to attitudes that may agree or disagree with paying attention to the category of patients’ pain [43, 44].However organizations can use the results of examining the attitudes and opinions of staff to make decisions and take specific measures and apply them to new plans and methods for pain management and control [42].

Despite the importance and emphasis on pain management and control, few clinical studies have been conducted in this area in Iran, and the issue of pain has received little attention in both medical and nursing opinions [45, 46]. Unfortunately, most studies conducted in this field indicate a lack of attitude and knowledge of medical staffs and nurses [18, 20–23]. In the current study, 39.1% of the nurses had already participated in pain management and control training courses. Furthermore, there was significant association between nurses’ knowledge and attitudes and their training history. Our results were somehow in line with other similar studies in Iran [27, 47, 48]. These findings accentuate the importance of pain management and control training courses in this target population and can lead to significantly improvement of nurses’ knowledge and attitudes [41, 42, 49].

Our study didn’t show statistically significant relationship between nurses’ knowledge and their attitudes, while Hosseinzadegan et al., had the same results [48]. This issue may be in the context of not presenting practical topics in training courses or while studying. However, few studies, revealed a significant relationship between the knowledge and pain management and control attitude, although with a low correlation coefficient [22, 47, 50].

This study revealed that age had a significant impact on nurses’ attitude and knowledge, while older nurses had significantly less knowledge and more negative attitude toward pain control and management. Also, our results showed any significant relationship between gender and nurses’ attitudes and knowledge, which was similar to the results of Hosseinzadegan et al., and Mohammad Aliha et al., [47, 48], but different from some others [22, 50].

Education and work experience had a significant relationship with nurses’ attitude toward pain management and control, while it was found that nurses’ attitudes have become more negative with the increase of work experience. Also highly educated nurses had significantly more negative attitudes toward pain control and management. Our findings were contrary to some similar studies [47–51].There were discrepancies among study variables related to knowledge and attitudes, including health systems and cultural differences, as well as differences in the questionnaires used.

Although, interestingly, none of the 400 participants in this study answered question 7of the Pain management Knowledge Questionnaire, which asked the participants about the extent of addiction to narcotic analgesics if they use them based on a schedule and regularly. Lack of sufficient knowledge in this field can lead to under-medicated patients and reduce their quality of life.

## Conclusion

The current study revealed that pain management and control knowledge in most emergency nurses in the province was low, and most of them had a moderate attitude. However, there was a significant relationship between nurses’ knowledge and attitude and age and previously participation in training courses. We need more scientific and comprehensive pain management and control educational programs to improve knowledge and attitude among health workers and nurses.

Also, the study of factors affecting nurses’ level of knowledge and attitude in studies has had heterogeneous results, making it challenging to conclude in this area and clarifying the need for more extensive studies and even systematic reviews. However the generalization capacity of this study about knowledge and attitude regarding pain management and control may enhance to any other similar countries such as planned replication, sampling strategies, systematic reviews, reflection and higher-order conceptualization, thick description, mixed methods research, and multicenter studies.

## Data Availability

The datasets analyzed and presented in this study are available from the corresponding authors on reasonable request. Data are openly available in a public repository that issues datasets with the responsibility of the corresponding author. The data was not taken from publicly available database.
